# A Novel Naphthoquinone-Coumarin Hybrid That Inhibits BCR-ABL1-STAT5 Oncogenic Pathway and Reduces Survival in Imatinib-Resistant Chronic Myelogenous Leukemia Cells

**DOI:** 10.3389/fphar.2018.01546

**Published:** 2019-01-09

**Authors:** Patricia Martín-Rodríguez, Borja Guerra, Idaira Hueso-Falcón, Haidee Aranda-Tavío, Juan Díaz-Chico, José Quintana, Francisco Estévez, Bonifacio Díaz-Chico, Angel Amesty, Ana Estévez-Braun, Leandro Fernández-Pérez

**Affiliations:** ^1^Laboratorio de Farmacología Molecular y Traslacional, Instituto Universitario de Investigaciones Biomédicas y Sanitarias, Universidad de Las Palmas de Gran Canaria, Las Palmas, Spain; ^2^Departamento de Química Orgánica, Instituto Universitario de Bio-Orgánica Antonio González, Universidad de La Laguna, San Cristóbal de La Laguna, Spain; ^3^Laboratorio de Bioquímica, Instituto Universitario de Investigaciones Biomédicas y Sanitarias, Universidad de Las Palmas de Gran Canaria, Las Palmas, Spain

**Keywords:** leukemia, imatinib, BCR-ABL1, drug resistance, naphthoquinone, coumarin

## Abstract

BCR-ABL1-STAT5 is an oncogenic signaling pathway in human chronic myelogenous leukemia (CML) and it represents a valid target for anti-CML drug design. Resistance to direct BCR-ABL1 inhibitors is a common clinical issue, so STAT5 inhibition has become an interesting alternative target. In this study, the effects of NPQ-C6, a novel naphtoquinone-coumarin conjugate, were evaluated on human CML-derived K562 cells. Live-Cell Imaging analysis revealed that NPQ-C6 inhibited 2D (IC50_AUC_ = 1.4 ± 0.6 μM) growth of CML cells. NPQ-C6 increased sub-G1 and reduced G0/G1 cell cycle phases in a dose- and time-dependent manner. This effect on cell cycle was related to increased levels of apoptotic nuclei, cleavage of caspase-3, -9, and PARP and annexin V-positive cells. NPQ-C6 increased γH2AX, a double-strand DNA break marker. NPQ-C6 showed a wide range of modulatory effects on cell signaling through an early increased phosphorylation of JNK, P38-MAPK and AKT, and decreased phosphorylation of ERK1/2, BCR-ABL1, and STAT5. NPQ-C6 inhibited expression of c-MYC and PYM-1, two target gene products of BCR-ABL1/STAT5 signaling pathway. Cytokine-induced activation of STAT5/STAT3-dependent transcriptional and DNA binding activities were also inhibited by NPQ-C6. Notably, NPQ-C6 maintained its activity on BCR-ABL1/STAT5/c-MYC/PIM-1 oncogenic pathway in imatinib-resistant cells. Molecular modeling suggested BCR-ABL1 and JAK2 proteins as NPQ-C6 targets. In summary, our data show a novel multikinase modulator that might be therapeutically effective in BCR-ABL1-STAT5-related malignancies.

## Introduction

Human chronic myelogenous leukemia (CML) is a hematological stem cell disorder characterized by excessive proliferation of cells of the myelogenous lineage (Ren, 2005). The main mark of CML is the Philadelphia chromosome, result of a genetic translocation that originates a fusion gene whose protein product is the chimerical BCR-ABL1 tyrosine kinase which is constitutively active. This oncoprotein is found in 95% of patients with CML and in approximately 5–10% of adults with acute leukemia without evidence of antecedent CML (Ren, 2005). BCR-ABL1 modulates intracellular signal pathways involved in proliferation (activation), apoptosis (inhibition) and cellular adhesion mechanisms (debilitation) (Ren, 2005; McCubrey et al., 2008; Sinclair et al., 2013). Activation of STAT5 by BCR-ABL1 induces downstream signaling pathways related to augmented expression of genes implicated in cell cycle progression, promoting survival and oncogenesis. Furthermore, STAT5 activity is associated with poor prognosis in CML and its deletion in BCR-ABL1^++^ cells induces apoptosis, even in cells which have developed resistance to tyrosine kinase inhibitors (TKI) (Ren, 2005).

Imanitib mesylate (IM), the accepted first-line therapy for all CML patients, is an ATP-competitive selective inhibitor of BCR-ABL1 (Quintas-Cardama et al., 2009). However, despite the initial successful response to this drug, IM fails in up to 40% of patients due to unacceptable side effects or developing resistance. This issue asks for alternative therapies to treat CML patients (Druker et al., 2006; Bixby and Talpaz, 2011; Warsch et al., 2011). Second generation TKIs (i.e., nilotinib, dasatinib) may provide long-term disease control but these drugs also have significant potential toxicities (Quintas-Cardama et al., 2009). Multifactorial events for IM resistance have been described, including mutations affecting the kinase domain of BCR-ABL1, which results in the inability of TKIs to bind to the kinase domain, or affecting its activity, as well as increased expression of BCR-ABL1, alterations of drug efflux-influx pump and increased STAT5 activity (McCubrey et al., 2008; Quintas-Cardama et al., 2009; Bixby and Talpaz, 2011; Quintas-Cardama and Verstovsek, 2013; Dorritie et al., 2014). Importantly, CML cells with mutant BCR-ABL1 presenting resistance to TKIs, show similar sensitivity to STAT5 inhibitors as cells with unmutated BCR-ABL1 (Nelson et al., 2011; Bar-Natan et al., 2012). Thus, inhibition of pY-STAT5 constitutes an attractive target in blood cancer, in general, and to overcome resistance to clinically used TKI in CML, in particular (Nelson et al., 2011; Bar-Natan et al., 2012). Notably, preclinical and clinical studies suggest that the combination of TKIs with multikinase inhibitors might be positive in comparison to selective TKIs because oncogenesis and drug resistance may be related to activation of alternative BCR-ABL1 mitogenic signals (Dufies et al., 2011).

The naphthoquinone (NPQ)-based derivatives are privileged chemical structures commonly used to generate antitumor agents (Hsu et al., 2006; Zhao et al., 2015; Guerra et al., 2017). Some quinone-antitumor agents have been used to treat solid tumors (e.g., doxorubicin) or acute lymphoblastic and myeloblastic leukemias (e.g., daunorubicin). NPQ-based derivatives exhibit pharmacological activities by a number of mechanisms, including oxidative stress, Michael-type arylation of thiol groups in biomolecules, DNA intercalation and bioreductive alkylation *via* quinone methide formation, autophagy, inhibition of topoisomerases, cell cycle arrest, apoptosis, or inhibition of c-MYC and BCR-ABL1/STAT5 pathway (Hsu et al., 2006; Zhao et al., 2015; Guerra et al., 2017; Hueso-Falcon et al., 2017). Coumarins are also considered as privileged chemical structures which exhibit a wide range of biological effects, including anticancer activities, generally associated with low toxicity (Medina et al., 2015). Recently, it has been shown that coumarin-chalcone hybrids are able to reduce cell growth and induce apoptosis in K562 cells (Elshemy and Zaki, 2017). Therefore, NPQ and coumarin represent promising scaffolds in medicinal chemistry for finding novel inhibitors of carcinogenic pathways. This is exemplified by the discovery of NPQ-coumarin hybrids as inhibitors of topoisomerase II (Hueso-Falcon et al., 2017). In this study, we report the NPQ-coumarin hybrid compound 7-(3,4-dimethoxyphenyl)-6H,7H-benzo[h]chromeno[4,3-b]chromene-6,8,9-trione (NPQ-C6) as a unique inhibitor BCR-ABL1-STAT5 oncogenic pathway that was effective against IM-resistant CML cells. These findings provide new insights into molecular mechanism of NPQ-coumarin conjugates in cancer and support its potential therapeutic application in BCR-ABL and STAT5-related malignancies.

## Materials and Methods

### Synthesis of NPQ-C6

7-(3,4-dimethoxyphenyl)-6*H*,7*H*-benzo[*h*]chromeno[4,3-*b*]chro mene-6,8,9-trione (NPQ-C6) was synthesized through a straightforward, one-protocol based on a three-component reaction with 2-hydroxy-1,4-naphthoquinone, 4-hydroxycoumarin, and 3,4-dimethoxybenzaldehyde as synthetic inputs, using InCl_3_ a catalyst under solvent-free conditions. Thus, 53.9 mg of 2-hydroxy-1,4-naphthoquinone (0.30 mmol), 51.2 mg of 3,4-dimethoxybenzaldehyde (0.31 mmol) and 49.2 mg of 4-hydroxycoumarin (0.30 mmol) were grinded in a mortar for 5 min. Then 21.1 mg of InCl_3_ (30 mol %) was added and the reaction mixture was grinded again for 15 min, placed in a sealed tube and kept in an oven at 120°C for 4 h. The resulting crude was purified by preparative-TLC with dichloromethane as solvent to afford 54.5 mg of NPQ-C6 as an amorphous orange solid. ^1^H-NMR (δ, 400 MHz, CDCl_3_): 8.16 (1H, dd, *J* = 7.7, 1.3 Hz, H-10), 8.14 (1H, dd, *J* = 7.7, 1.3 Hz, H-13), 8.07 (1H, dd, *J* = 8.2, 1.5 Hz, H-1), 7.85 (1H, td, *J* = 7.7, 1.3 Hz, H-12), 7.65 (2H, m, H-3, H-11), 7.46 (1H, td, *J* = 8.2, 1.0 Hz, H-2), 7.39 (1H, dd, *J* = 8.2, 1.0 Hz, H-4), 7.16 (1H, d, *J* = 2.1 Hz, H-2′), 6.77 (1H, dd, *J* = 8.4, 2.1 Hz, H-6′), 6.70 (1H, d, *J* = 8.4 Hz, H-5′), 5.13 (1H, s, H-7), 3.90 (3H, s, H-1″), 3.77 (3H, s, H-2″); ^13^C-NMR (δ, 100 MHz, CDCl_3_): 178.2 (s, C-8), 177.4 (s, C-9), 160.3 (s, C-6), 155.4 (s, C-13b), 153.6 (s, C-14a), 152.9 (s, C-4a), 149.1 (s, C-3′), 148.7 (s, C-4′), 135.6 (d, C-12), 133.9 (s, C-1′), 132.9 (d, C-3), 132.0 (d, C-11), 130.3 (d, C-10), 130.1 (s, C-9a), 130.1 (s, C-13a), 124.8 (d, C-2), 124.4 (d, C-13), 122.3 (d, C-1), 120.2 (d, C-6′), 117.4 (d, C-4), 117.4 (s, C-6a or C-7a), 113.6 (s, C-14b), 113.4 (d, C-2′), 111.4 (d, C-5′), 106.7 (s, C-6a or C-7a), 56.2 (q, C-1″), 56.0 (q, C-2″), 33.4 (d, C-7); HRMS-ESI (+): 489.0945 (calc for C_28_H_18_O_7_Na [M+23(Na)]^+^ 489.0950); IR 𝒱_max_ 3083, 2935, 2837, 1725, 1657, 1605, 1589, 1513, 1456, 1420, 1358, 1263, 1236, 1188, 1138, 1104, 1083, 1050, 1024, 947, 869, 828, 769, 708, 648 cm^-1^.

### Reagents and Antibodies

Z-VAD was purchased from Calbiochem (San Diego, CA, United States). Necrostatin-1 and 3-methyladenine (3-MA) were purchased from Sigma-Aldrich (St. Louis, MO, United States). RPMI-1640, DMEM, McCoy’s 5A, fetal bovine serum (FBS), L-glutamine and PEST (50 units/ml penicillin, 50 μg/ml streptomycin) were obtained from Lonza (Belgium). Recombinant human Erytropoyetin (hEPO) and GH were kindly provided by Roche and Pfizer Spain laboratories, respectively. Oncostatin M (OSM) was supplied by Miltenyi Biotec (Gladbach, Germany) and HumanZyme (Chicago, IL, United States), respectively. The anti-CML drug IM (Quintas-Cardama et al., 2009) was purchased from Calbiochem (San Diego, CA, United States). Monoclonal and polyclonal antibodies used in the Western blotting analyzes were as follows: pTyr^694^-STAT5 (pYSTAT5), pTyr^705^-STAT3 (pYSTAT3), pTyr^1007/1008^JAK2 (pYJAK2), pTyr^177^-BCR (pYBCR-ABL1/pYBCR), pThr^183^/Tyr^185^-JNK (pJNK), pSer^473^-AKT (pSer-AKT), pThr^308^-AKT (pThr-AKT), pThr^202^/pTyr^204^-ERK1/2 (pERK1/2), BCR, PIM-1, AKT, ERK1/2, JAK2, and STAT3 were obtained from Cell Signaling Technology (Leiden, Netherlands). Antibodies against β-actin, STAT5, JNK1/3 (C-17), c-MYC, and the horseradish peroxidase-conjugated secondary antibodies goat anti-rabbit and goat anti-mouse were provided by Santa Cruz Biotech (Santa Cruz, CA, United States). Antibodies to caspase-3, -8, and -9 were obtained from Enzo Life Sciences (Lausen, Switzerland). Antibody against PARP was obtained from BD Biosciences (Erembodegem, Belgium). Antibody against γH2AX was obtained from BioLegend (London, United Kingdom). Enhanced chemiluminescent detection system was provided by Bio-Rad (Munch, Germany). Other generic chemicals cited in this work were supplied by Sigma-Aldrich, Roche Biochemicals (Mannheim, Germany), or Merck (Darmstadt, Germany).

### Cells

The cell lines were growth at 37°C under 5% CO_2_ under humidified atmosphere. All cell lines were purchased from the American Type Culture Collection (ATCC). The human cell lines K562 (CML), HEL (erythroleukemia), HL60 (acute myeloid leukemia), MOLM.13 (acute myeloid leukemia), MV4.11 (acute monocytic leukemia), PC3 (prostate cancer), HCT-15 (colorectal adenocarcinoma), BT-549 (breast cancer), MCF7 (breast cancer), MRC.5 cells (non-malignant lung fibroblasts) and non-human cell lines NCTC3749 (mouse lymphoma) were grown in RPMI-1640 medium. The K562-R cell line, a CML resistant to IM obtained by subculturing K562 in a steplike arrangement of increasing concentration of IM (Guerra et al., 2017), was maintained in RPMI-1640. The human cell lines HeLa (epithelial cervix cancer), MDA-MB-231 (breast cancer), HS-578T (breast cancer) and T47D (breast cancer adenocarcinoma) and non-human cell lines L1210 (mouse lymphocytic leukemia cells), Vero (monkey non-malignant kidney cells) and Raw (mouse macrophage), were grown in DMEM. HEK293 cells stably expressing the GH receptor (HEKGHR) were maintained in DMEM-F12 medium (Maamra et al., 1999). SKBR3 (human breast cancer) cell line was maintained in McCoy’s 5A medium. Human peripheral blood mononuclear cells (PBMCs) were isolated from EDTA-anticoagulated blood of healthy volunteers by centrifugation with Ficoll-Paque^TM^ PLUS (GE Healthcare Bio-Sciences AB; Uppsala, Sweden). PMBCs were maintained in RPMI-1640 medium. All of cell culture mediums were supplemented with 10% FBS, L-glutamine (2 mM) and PEST. In addition, the culture medium for T47D and BT-549 was supplemented with 1 mM NaPyr and 10 mM HEPES.

### Cell Viability Assay

Tumor and MRC.5 cells were seeded at exponential growth density (5000–10000 cells per well) in 96-well plates (BD Falcon, France). Cells were treated with vehicle (0.05% DMSO) or compound (0.01 to 10 μM) for 48 h. PMBCs were seeded (100000–200000 cells per well) in 96-weel plates (BD Falcon, France) and treated with vehicle (0.05% DOMSO), NPQ-C6 (1 and 3 μM) or doxorubicin (1 and 3 μM) for 24 h. After treatment period, the tetrazolium salt 3-(4,5-methyltiazol-2yl-)-2,5diphenyl-tetrazolium bromide (MTT) (AppliChem, Germany) was added and the cells were incubated for 2–4 h. The mitochondrial metabolization of the MTT was used as indicator of cell viability (Mosmann, 1983). Cells were lysed in 10% SDS and optical density was measured at 595 nm with the iMark Microplate Reader (BioRad).

### Real-Time Monitoring of Tumor Growth

Continuous monitoring of K562 cells was performed using the IncuCyte^TM^ HD real-time imaging system (Essen BioScience, United Kingdom). K562 cell proliferation and cytotoxicity were measuring after seeding 5000 cells per well at polylysine-coated 96 well-plate (Rainaldi et al., 1998) and monolayer allowed to form (18 h). Then, cells were treated with vehicle (0.05% DMSO) or serial compound dilutions (0.03–10 μM) in the presence of 25 nM of the live-cell imaging reagent YOYO-1 (Invitrogen). YOYO^®^-1 is a cell impermeant cyanine dimer nucleic acid stain that binds to dsDNA (Becker et al., 1994). YOYO^®^-1 fluorescently stains the nuclear DNA of cells that have lost plasma membrane integrity, which enables the kinetic detection of cytotoxicity. IncuCyte^TM^ microscope allowed the acquisition of automated phase contrast images. Individual images were processed by an imbedded contrast-based confluence algorithm, which computed monolayer confluence for each image and at each time point. Multiple images were collected per well and averaged to provide a representative statistical measure of confluence and cytotoxicity per well. Data were collected over 96-h period at 6-h intervals. The data were plotted as concentration-response [area under curve (AUC)] curves at 96-h post-treatment.

### Luciferase Reporter Gene Assay

The HEKGHR cell line, stably transfected with GHR, was used to determine the effects of compounds on GH-regulated STAT5 transcriptional activity (Maamra et al., 1999). Cells were seeded at a density of 400000 cells per well in 12-well culture plates for 24 h. HEKGHR cells were serum deprived and transfected with pSPI-GLE1-Luc (1 μg) overnight using Metafectene Pro^®^ (Biontex, Germany). The transfection medium was removed and the cells were treated with vehicle (0.05% DMSO) or compound (0.1–10 μM) in the absence of serum and for 1 h. Following pre-incubation with the drug, rhGH (50 nM) (Maamra et al., 1999) was added and cells were incubated for an additional 6 h. The stable reporter cell line HeLa/STAT3-luc (Panomics, United States), was used to determine the effects of compounds on Oncostatin M (OSM, Humanzyme, United States)-regulated STAT3 transcriptional activity (Stephens et al., 1998). Cells were seeded at a density of 240000 cells per well in 6-well culture plates for 24 h and they were serum deprived (0.5% FBS) for 16 h. Then, cells were treated with vehicle (0.05% DMSO) or compound (0.1 to 10 μM) during 1 h before oncostatin M (50 ng/ml) were added for additional 6 h (Guerra et al., 2017). Cells were lysed in Passive Lysis Buffer (Promega, United States) and luciferase activity was determined by the luciferase assay system (Thermo Scientific, United States). Luciferase activity was measured using the microplate reader Fluoroskan Ascent FL (Labsystems). The results are expressed as relative luciferase units (RLUs) per mg protein and normalized by results obtained for vehicle-treated control cells.

### Cell Cycle Analysis and Evaluation of Apoptosis

Unsynchronized K562 cells were treated with vehicle (0.05% DMSO) or compounds in the presence FBS for 24 or 48 h at different concentrations (5, 10 μM) of drug. Then, cells were fixed in 70% ethanol and incubated with propidium iodide (PI) in the presence of RNAse. Nuclei to the left of the 2N peak containing hypodiploid DNA were considered as apoptotic. Fluorescent microscopy and flow cytometric analysis of PI-stained nuclei were performed to evaluate cell cycle and viability by using a FACSCalibur cytometer and CellQuest software (BD Biosciences, Belgium) (Guerra et al., 2017). Apoptosis was also determined by translocation of phosphatidylserine to the cell surface using the annexin V-FITC apoptosis detection kit (BD Pharmingen, Belgium) according to the manufacturer’s protocol. Annexin positive cells were considered as apoptotic while annexin and PI positive cells were considered as necrotic. Dyeing with the fluorochrome trihydrochloride of bisbenzimide Hoescht 33258 (Sigma-Aldrich, United States) was used to assess nuclear morphology. Briefly, 24 h after K562 cells were seeded (125000 cells/ml), they were treated with vehicle or NQP6 (5–10 μM) for 48 h. Then, K562 cells were PBS washed, fixed with 3% paraformaldehyde, and mixed with 20 μl Hoescht (16 μg/ml) in the dark for 15 min. Finally, cell suspension (10 μl) was analyzed under a fluorescence microscope to quantify and photograph the apoptotic nuclei.

### Assay of Caspase Activity

K562 cells were treated with vehicle (0.05% DMSO), NPQ-C6 (5 μM for 6 or 24 h) or etoposide (30 μM for 24 h). After treatments, K562 cells were harvested by centrifugation at 1000 × *g* for 5 min at 4°C and washed with PBS, and the cell pellets were kept on ice. The cells were resuspended in cell lysis buffer (50 mM HEPES, pH 7.4, 1 mM dithiothreitol, 0.1 mM EDTA, 0.1% Chaps) and held on ice for 5 min. After centrifugation for 10 min at 17,000 × *g* at 4°C, the supernatants were analyzed for protein concentration by the Bradford dye binding assay and stored at ∼20°C until used to study caspase colorimetric enzymatic activity. Equal amounts of protein (∼20 μg) from different treatments were used, and the assays were set up on ice. The net increase of absorbance at 405 nm after incubation at 37°C was indicative of enzyme activity. Specific labeled substrates for caspase-3, -8, and 9 activities were *N*-acetyl-Asp-Glu-Val-Asp-*p*-nitroaniline (DEVDpNA), *N*-acetyl-Ile-Glu-Thr-Asp-*p*-nitroaniline (IETD-pNA) and *N*-acetyl-Leu-Glu-His-Asp-*p*-nitroaniline (LEHD-pNA) (Calbiochem, San Diego, CA, United States), respectively.

### STAT-DNA Binding Activity Analysis

Cells were treated with vehicle (0.05% DMSO) or compounds in the absence FBS as indicated in figure legends. Then, the cells were stimulated with GH (50 nM) for 10 min or IL-6 (25 ng/ml) for 30 min. The cells were rinsed with cold PBS-orthovanadate (1 mM) followed by harvesting cytosolic and nuclear proteins, in the presence of phosphatase and protease inhibitor cocktail, by using a Nuclear Extract kit (Active Motif, Inc., United States) according to the protocol supplied by the manufacturer. STAT5- or STAT3-specific DNA-binding activities in nuclear extracts were measured by using a TransAM STAT kit (Active Motif, United States). Protein concentration was measured by the bicinchonin acid assay (Pierce^®^ BCA Protein Assay Kit; Thermo Scientific, United States). Absorbance was quantified with the iMark Microplate Reader (BioRad).

### Immunoblotting

Cells were treated with vehicle (0.05% DMSO) or compounds in the presence or absence FBS as indicated in figure legends. Then, cells were rinsed with cold PBS-orthovanadate (1 mM) centrifuged and then pellet was mixed with a suitable volume of RIPA (Pierce, United States), in the presence of protease and phosphatase inhibitors, for obtaining the total protein extract. Samples were allowed to stir gently for 30 min at 4°C, centrifuged at 18000 × *g*, 5 min and 4°C, and supernatant was taken. Amount of protein was quantified by the bicinchonin acid assay. Equal amounts of each sample were separated by SDS-PAGE and blotted onto nitrocellulose membranes. After being blocked with 1% BSA-1% Blotto (anti-phospho-antibodies) (Santa Cruz Biotech, United States) or 5% blotto (anti-total-antibodies) (Santa Cruz Biotech, United States) (both diluted in Tris buffered saline with 0.05% Tween 20), membranes were immunoblotted overnight at 4°C with dilution (1:1000) in the same buffer of blocking of primary phosphoprotein or total protein antibodies. Antibody-specific labeling was revealed by incubation with a HRP-conjugated goat anti-mouse secondary antibody (Santa Cruz Biotech, United States) or a HRP-conjugated goat anti-rabbit secondary antibody (Santa Cruz Biotech, United States) (1:5000) for 1 h and visualized with the Immu-Star^TM^ WesternC^TM^ kit (Bio-Rad, United States) using the ChemiDoc XRS system (Bio-Rad), and the image analysis program Quantity one (Bio-Rad). As charge and membrane transfer efficiency control, membranes were incubated with a monoclonal mouse anti-beta actin antibody (Santa Cruz Biotech, United States) (Guerra et al., 2011).

### Protein Preparation and Docking

The X-ray coordinates of the corresponding BCR-ABL1 and JAK2 kinase domain were extracted from the Protein Data Bank (PDB code 1IEP, 1FPU, 1M52, 3CS9, 2HYY, 5HU9, 3K5U, 3QRI, 2GQG, 5CF4, 3TJD, 3TJC, 3FUP, and 3KCK). The PDB structures were prepared for docking using the Protein Preparation Workflow (Schrödinger, 2017) accessible from within the Maestro program 11.4 (Maestro, 2017). The substrate and water molecules were removed beyond 5 Å, bond corrections were applied to the co-crystallized ligands and an exhaustive sampling of the orientations of groups was performed. Finally, the receptors were optimized in Maestro 11.4 (Maestro, 2017) by using OPLS3 force field before docking study. In the final stage the optimization and minimization on the ligand–protein complexes were carried out with the OPLS3 force field and the default value for rmsd of 0.30 Å for non-hydrogen atoms were used. The receptor grids were generated using the prepared proteins, with the docking grids centered on the center of the bound ligand for each receptor. A receptor grid was generated using a 1.00 van der Waals (vdW) radius scaling factor and 0.25 partial charge cutoff. The binding sites were enclosed in a grid box of 20 Å^3^ with default parameters and without constrains. The three-dimensional structures of the ligand to be docked was generated and prepared using LigPrep (LigPrep, 2017) as implemented in Maestro 11.4 (Maestro, 2017) to generate the most probable ionization states at pH = 7 ± 1 (retain original ionization state). These conformations were used as the initial input structures for the docking. In this stage a series of treatments are applied to the structures. Finally, the geometries are optimized using OPLS3 force field. These conformations were used as the initial input structures for the docking. The ligands were docked using the extra precision mode (XP) (Friesner et al., 2006) without using any constraints and a 0.80 van der Waals (vdW) radius scaling factor and 0.15 partial charge cutoff. The dockings were carried out with flexibility of the residues of the pocket near to the ligand. The generated ligand poses were evaluated with empirical scoring function, GlideScore, a modified version of ChemScore (Eldridge et al., 1997). GlideScore implemented in Glide, was used to estimate binding affinity and rank ligands (Friesner et al., 2004). The XP Pose Rank was used to select the best-docked pose for each ligand. The best correlation with the BCR-ABL1 and JAK2 inhibition and the best values of docking score were achieved when the 2GQG and 5CF4 were used.

### Statistical Analysis

Results represent data obtained from at least three independent experiments, each performed in triplicate (mean ± SEM). The significance of differences between groups was tested by one-way ANOVA, followed by *post hoc* comparisons of group means or Student’s *t*-test according to the GraphPad Prism 5 program^1^. Statistical significance was reported if *p* < 0.05 was achieved. The concentration required to reduce cell viability/proliferation by 50% (IC50) was determined graphically using the curve fitting algorithm of the GraphPad.

## Results

### NPQ-C6 Reduces Viability of Human Leukemia Cells

**FIGURE 1:**
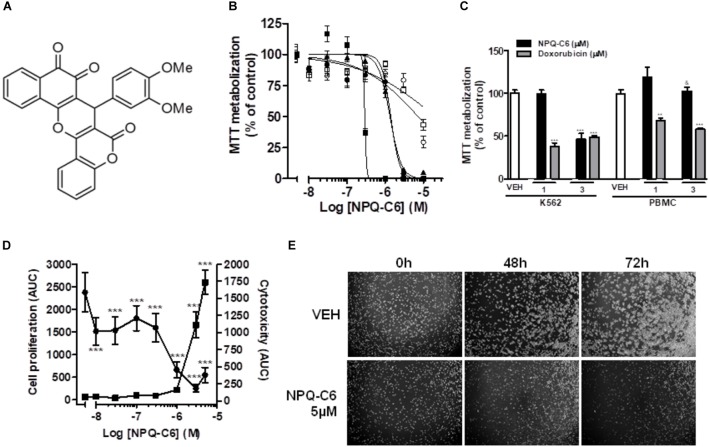
NPQ-C6 reduces viability and growth of human leukemia cells. **(A)** Chemical structure of NPQ-C6. **(B)** Cells K562 (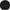), HL60 (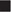), HEL (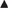), MRC5 (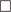), and Vero (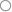) were cultured in the presence of increasing concentrations (0.01–10 μM) of NPQ-C6 for 48 h. Subsequently, cell viability was determined by the MTT assay. **(C)** PBMC were cultured in the presence of different concentrations (1 and 3 μM) of NPQ-C6 for 24 h. Subsequently, cell viability was determined by the MTT assay. **(D)** K562 cells were cultured in the presence of vehicle (VEH) or increasing concentrations of NPQ-C6 (0.01–10 μM) for 4 days. The effects of NPQ-C6 on cell proliferation (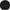) and cytotoxicity (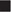) were investigated using the Incucyte^TM^ HD real-time analysis system. Data are represented as area under the curve (AUC). **(E)** Representative photomicrographs of exponentially growing K562 cells in the absence (vehicle) or presence of NPQ-C6 for 48 and 72 h. Figures are representative of three independent experiments each one performed in triplicate. ^∗^*P* < 0.05; ^∗∗^*P* < 0.01; ^∗∗∗^*P* < 0.001 versus vehicle-treated cells (VEH). ^&^*P* < 0.05 versus K562 cells.

NPQ-C6 was discovered by high-throughput cell based phenotypic screening of a proprietary small molecule library (Figure 1A). Firstly, viability of exponentially growing cells of different hematological and solid tumors was determined by using the MTT metabolization assay in the presence of NPQ-C6 for 48 h. NPQ-C6 showed higher antitumoral potency on leukemia cells (i.e., HL60, MOLM.13, MV4.11, HEL, K562) than on non-hematological (i.e., MCF7, SKBR3, MDA-MB 231) and non-malignant (i.e., MRC.5 and Vero) cells (Figure 1B and Table 1). Moreover, we investigated whether NPQ-C6 was also cytotoxic for human PMBCs. Cell viability of quiescent PMBC remained unaffected (up to 3 μM) but was significantly reduced in exponentially growing-K562 cells or proliferating PMBC after NPQ-C6 treatment for 24 h (Figure 1C). However, doxorubicin (1 and 3 μM for 24 h), a drug clinically used against several types of cancer including leukemia, caused a similar reduction on K562 and quiescent PMBC cell viability (Figure 1C). Secondly, *in vivo* Live-Cell Imaging of K562 cells demonstrated that NPQ-C6 caused a cytostatic effect on K562 cells growth at a concentration close to 0.7 μM and a cytotoxic effect at concentrations higher than 1.7 μM (Figure 1D). As expected (Levitzki, 2012), IM caused a cytostatic effect on K562 cells (IC50_AUC_ = 0.2 ± 0.1 μM) (data not shown). Photomicrograph of each well-supported the effects of 5 μM NPQ-C6 on K562 cells (Figure 1E). Taken together, these results suggested that CML cell growth is acutely sensitive to NPQ-C6.

**Table 1:** Effects of NPQ-C6 on blood and non-blood cancer cell viability.

Cell line	IC50 (mean ± *SD*)
HL60	0.3 ± 0.1
MOLM.13	0.5 ± 1.1
MV4.11	1.1 ± 1.1
HEL	1.3 ± 0.6
K562	1.4 ± 0.7
T47D	2.1 ± 1.2
PC3	3.2 ± 1.1
NCTC 3749	3.4 ± 1.6
L1210	4.7 ± 1.1
HCT-15	4.8 ± 1.4
BT-549	4.8 ± 1.4
MCF7	8.3 ± 0.1
MDA-MB 231	>5
HS-578T	>5
MRC-5	>5
PBMC^∗^	4 ± nd
Vero	6.1 ± 1.5


*Cells were treated with vehicle (0.05% DMSO) or NPQ-C6 (0.01 to 10 μM) for 48 h. Cell viability was assessed by MTT assay as described in Section “Materials and Methods.” ^∗^Cells were treated with vehicle or NPQ-C6 for 24 h.*

### NPQ-C6 Blocks Cell Cycle Progression in Human Chronic Myelogenous Leukemia Cells

**FIGURE 2:**
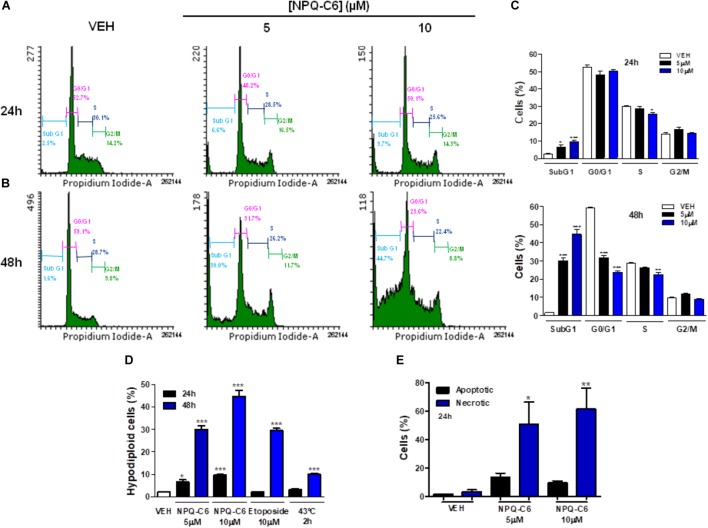
NPQ-C6 blocks cell cycle progression in human chronic myelogenous leukemia (CML) cells. K562 cultures were treated with different doses of NPQ-C6 (5 and 10 μM) for **(A)** 24 – **(B)** 48 h. **(C)** Subpopulations of cells were quantified as percentage of cells in the sub-regions G0, M o S by fluorescence flow cytometry as described in Section “Materials and Methods.” Figures are representative of two independent experiments each one performed in triplicate. **(D)** K562 cells were treated with vehicle (0.1% DMSO) or compounds (NPQ-C6 5 and 10 μM or etoposide 10 μM) for 24–48 h. Then, cells were fixed in 70% ethanol and incubated with propidium iodide (PI) in the presence of RNAse. Nuclei to the left of the 2N peak containing hypodiploid DNA were considered as apoptotic. **(E)** K562 cells were treated with vehicle (0.1% DMSO) or NPQ-C6 (5 and 10 μM) for 24 h and apoptosis was determined by translocation of phosphatidylserine to the cell surface using the annexin V-FITC apoptosis detection kit as described in Section “Materials and Methods.” Data of apoptotic and necrotic cells are shown. Figures are representative of two independent experiments each one performed in triplicate. ^∗^*P* < 0.05; ^∗∗^*P* < 0.01; ^∗∗∗^*P* < 0.001 versus vehicle-treated cells (VEH).

**FIGURE 3:**
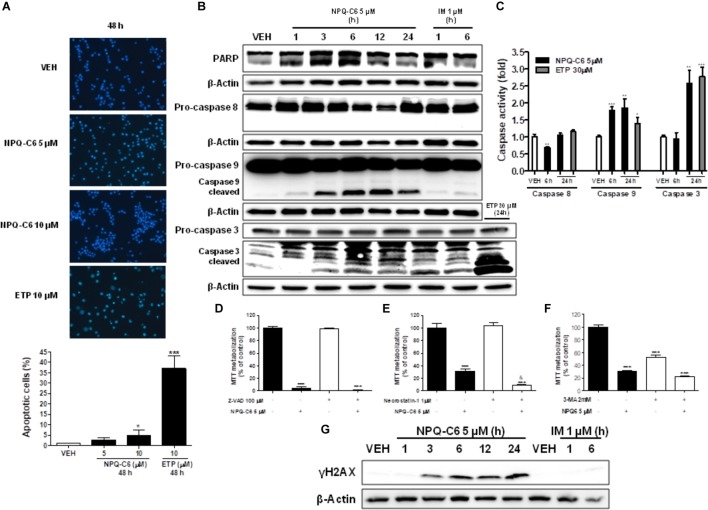
NPQ-C6 induces apoptosis in K562 cells. **(A)** Photomicrographs of representative fields of K562 cells stained with bisbenzimide trihydrochloride to evaluate nuclear chromatin condensation after treatment for 48 h with NPQ-C6 (5 and 10 μM) or etoposide (10 μM). **(B)** K562 cultures were incubated with vehicle, NPQ-C6 (5 μM), imatinib (IM) (1 μM), or etoposide (ETP) (30 μM) for indicated times. Then, whole cell extracts were obtained and proteins related to apoptosis (PARP and pro-caspases 3, -8, and -9) were analyzed by immunoblotting. **(C)** Kinetics of caspase-3, -8, and -9 activation in response to NPQ-C6 or etoposide. K562 cells were treated with 5 μM NPQ-C6 or 30 μM etoposide and harvested at the indicated times. Cell lysates were assayed for caspase-3, -8, and -9 activities using the DEVD-pNA, IETD-pNA, and LEHD-pNA colorimetric substrates, respectively. Results are expressed as fold increase in caspase activity compared with vehicle (0.05% DMSO). K562 cells were cultured in the presence of **(D)** inhibitor of caspases Z-VAD (100 μM), **(E)** Necrostatin-1 (1 μM) and **(F)** 3-Methyladenine (2 mM) for 1 h 37°C. Then, vehicle or NPQ-C6 5 μM was added for 24 h. Subsequently, cell viability was determined by the MTT assay. **(G)** K562 cultures were incubated with vehicle, NPQ-C6 (5 μM) or imatinib (IM) (1 μM) for indicated times. Then, whole cell extracts were obtained and protein content of the double-strand DNA break marker γH2AX was analyzed by immunoblotting. Figures are representative of two independent experiments each one performed in duplicate. ^∗^*P* < 0.05; ^∗∗^*P* < 0.01; ^∗∗∗^*P* < 0.001 versus vehicle-treated cells (VEH). ^&^*P* < 0.05 versus NPQ-C6-treated cells.

To investigate if the decrease of the K562 cell growth induced by NPQ-C6 was associated to cell cycle blockade, augmented cytotoxicity, or both, K562 cells were treated with NPQ-C6 (5 and 10 μM) for 24–48 h and cell cycle profiles and apoptotic induction were analyzed. NPQ-C6 caused a time and dose-dependent increase in sub-G1 phase and a reduction in G0/G1 (Figures 2A–C). NPQ-C6 induced a concentration- and time-dependent induction of apoptosis as determined by an increase in hypodiploid DNA content (Figure 2D) and translocation of phosphatidylserine to the cell surface (Figure 2E). In addition, NPQ-C6 reduced viability of K562 cells was associated with increased number of apoptotic nuclei (Figure 3A) and a time-dependent induction of the cleavage of caspase-3, -9, and poly(ADP-ribose) polymerase (PARP) (Figure 3B), as well as caspase-3, -9 activities (Figure 3C). However, neither the pan-caspases inhibitor Z-VAD (100 μM) (Figure 3D), nor necrostatin (1 μM), an inhibitor of necroptosis (Figure 3E) or 3-MA (2 mM), an inhibitor of autophagy (Figure 3F), prevented NPQ-C6-induced reduction of cell viability. Importantly, NPQ-C6 increased, in a time dependent manner, the double-strand DNA break marker γH2AX which suggests that K562 cells cannot overcome NPQ-C6-induced cytotoxicity and that they are destined for apoptosis (Figure 3G).

### NPQ-C6 Inhibits the BCR-ABL1-STAT5 and Induces JNK, p38-MAPK, and AKT Signaling Pathways in Human Chronic Myelogenous Leukemia Cells

**FIGURE 4:**
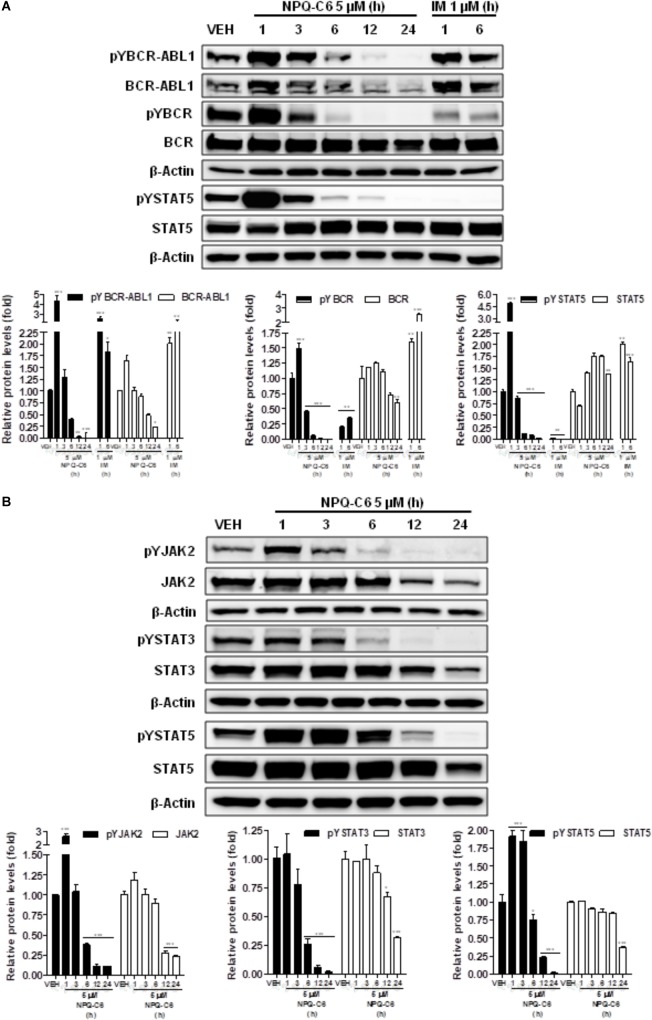
NPQ-C6 inhibits constitutive activation of BCR-ABL1-STAT5 and JAK2-STAT5 signaling pathways. **(A)** K562 cells were incubated in the presence of vehicle, NPQ-C6 (5 μM) or imatinib (IM) (1 μM) for the indicated times. Then, immunoblotting analyses were performed to detect the phosphorylated (pY) and total levels of BCR-ABL, BCR, and STAT5. Densitometric quantification of immunosignal values of phospho and total proteins relative to β-Actin (VEH: fold 1) are shown. **(B)** HEL cells were incubated in the presence of vehicle or NPQ-C6 (5 μM) for the indicated times. Then, immunoblotting analyses were performed to detect the phosphorylated (pY) and total levels of JAK2, STAT3 and STAT5. Densitometric quantification of immunosignal values of phosphor and total proteins relative to β-Actin (VEH: fold 1) are shown. Figures are representative of two independent experiments each one performed in duplicate. ^∗^*P* < 0.05; ^∗∗^*P* < 0.01; ^∗∗∗^*P* < 0.001 versus vehicle-treated cells (VEH).

**FIGURE 5:**
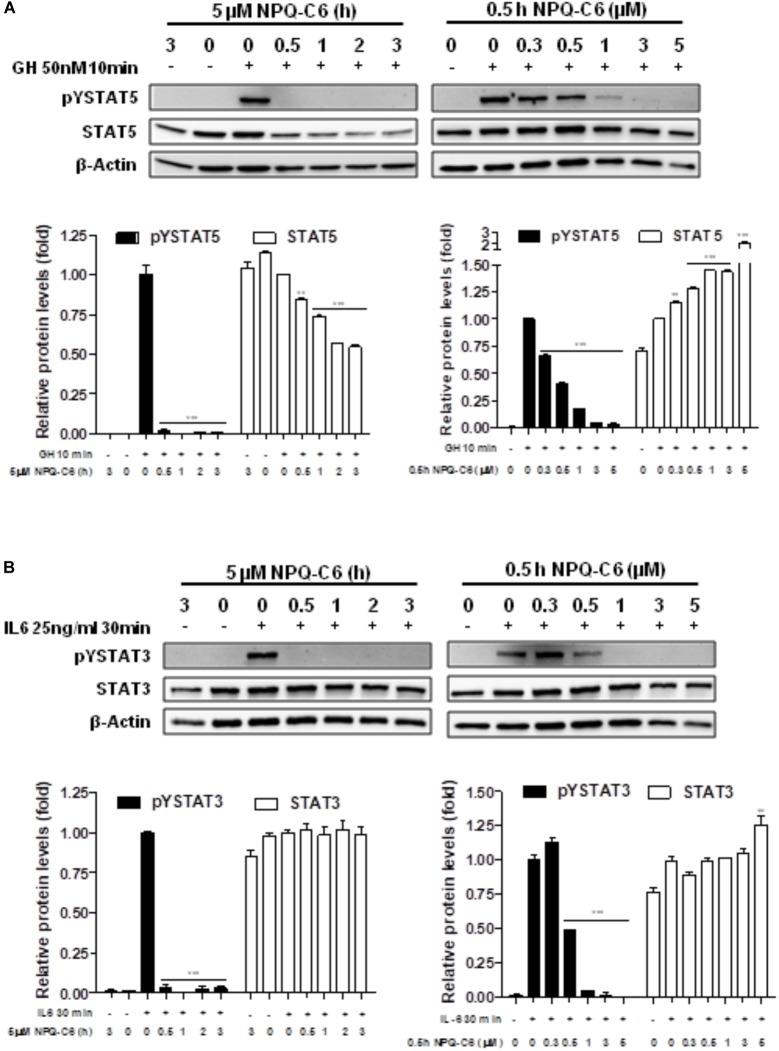
NPQ-C6 inhibits cytokine-induced tyrosine phosphorylation of STAT5 and STAT3. **(A)** Serum-deprived T47D cells were preincubated with NPQ-C6 (5 μM) for indicated times (**upper left panel**) or treated with different doses of NPQ-C6 (0–5 μM) for 30 min (0.5 h) (**upper** **right panel**). Then, T47D cells were stimulated with growth hormone (GH) (50 nM) for 10 min followed by immunoblotting analyses of phosphorylated (pY) and total STAT5 levels. Densitometric quantification from immunosignal values of phospho and total STAT5 relative to β-Actin (VEH: fold 1) are shown (**lower left and right panel**). **(B)** Serum-deprived T47D cells were preincubated with NPQ-C6 (5 μM) for indicated times (**upper left panel**) or treated with different doses of NPQ-C6 (0–5 μM) for 30 min (0.5 h) (**upper** **right panel**). Then, T47D cells were stimulated with interleukin-6 (IL-6) (25 ng/ml) for 30 min followed by immunoblotting analyses of phosphorylated (pY) and total STAT3 levels. Densitometric quantification from immunosignal values of phospho and total STAT3 relative to β-Actin (VEH: fold 1) are shown (**lower left and right panel**). Figures are representative of two independent experiments each one performed in duplicate. ^∗∗^*P* < 0.01; ^∗∗∗^*P* < 0.001 versus GH **(A**) or IL6 **(B)**-stimulated cells.

**FIGURE 6:**
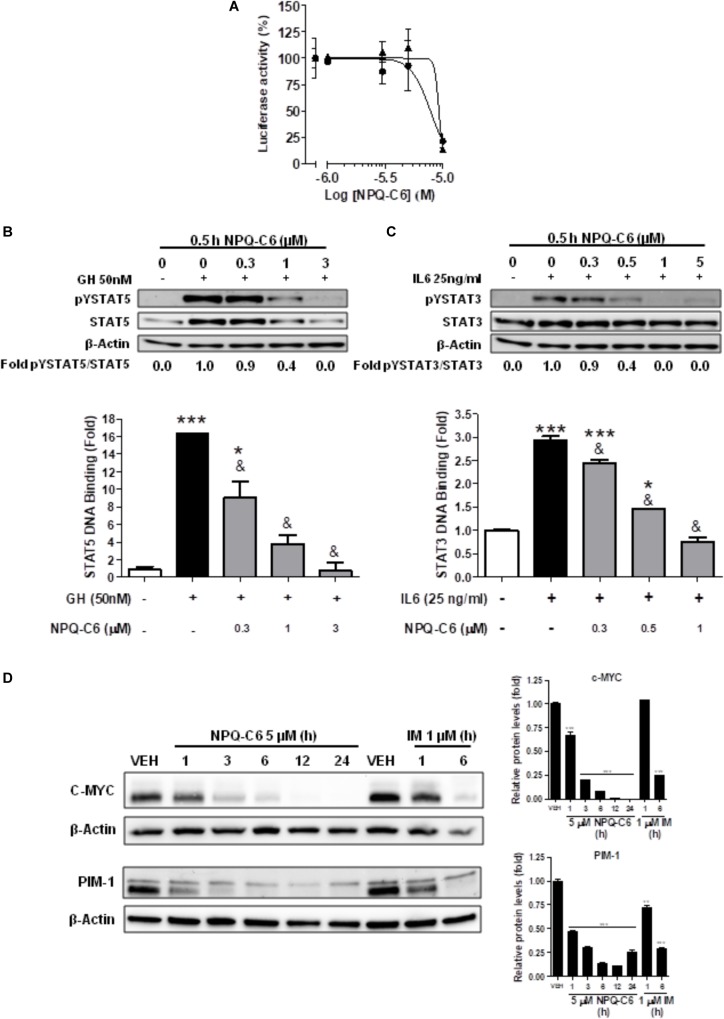
NPQ-C6 inhibits STAT-mediated cytokine-induced transcription, STAT-DNA binding activities and STAT-mediated C-MYC and PIM-1 protein expression. **(A)** Serum starved HEKGHR and HeLa/Stat3-luc cells were used to determine the effects of NPQ-C6 on on STAT5 (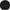) and STAT3 (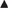) response element driving expression of a luciferase reporter gene, respectively. The expression vector for β-galactosidase protein was used to control transfection efficiency (data not shown). Then, cells were pretreated with vehicle or NPQ-C6 for 1 h followed by GH (for STAT5) or IL6 (for STAT3) for 6 h. Luciferase activity was measured as described in Section “Materials and Methods.” Serum-deprived T47D cells were pre-incubated for 30 min with NPQ-C6 (5 μM) and subsequently stimulated with GH (50 nM) for 10 min **(B)** or IL-6 (25 ng/ml) for 30 min **(C)**. Then, nuclear protein extracts were assayed by immunoblotting to detect the phosphorylated and total levels of STAT5 **(B; upper panel)** and STAT3 **(C; upper panel)**, respectively. β-Actin was used as a loading control. Nuclear proteins from NPQ-C6 and GH/IL-6-treated T47D cells were analyzed to measure the binding of STAT5b **(B; lower panel)** or STAT3 **(C; lower panel)** to DNA by using ELISA as described in Section “Materials and Methods.” **(D)** K562 cells were incubated in the presence of vehicle (VEH), NPQ-C6 (5 μM) or imatinib (IM) (1 μM) for the indicated times. Then, immunoblotting analyses were performed to detect C-MYC and PIM-1 protein levels. Densitometric quantification of immunosignal values of C-MYC and PIM-1 relative to β-Actin (VEH: fold 1) are shown. Figures are representative of 2–3 independent experiments each one performed in duplicate. ^∗^*P* < 0.05; ^∗∗^*P* < 0.01; ^∗∗∗^*P* < 0.001 versus vehicle-treated cells (VEH). ^&^*P* < 0.01 versus GH **(B)** or IL6 **(C)** stimulated cells.

**FIGURE 7:**
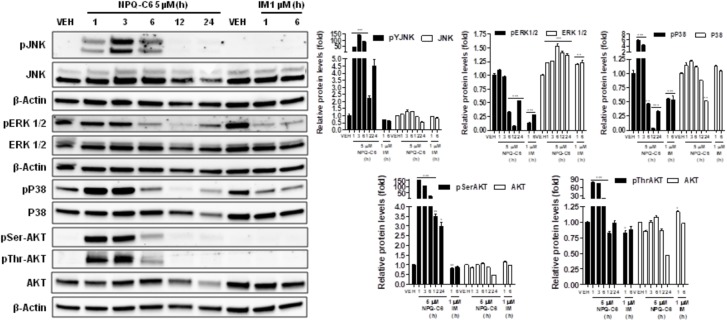
NPQ-C6 modulates survival signaling pathways. K562 cultures were incubated with vehicle (VEH), NPQ-C6 (5 μM) or IM (1 μM) for indicated times. Then, immunoblotting analyzes were performed to detect the phosphorylated and total levels of JNK, ERK1/2, P38-MAPK, and AKT. Densitometric quantification of immunosignal values of phosphor and total proteins relative to β-Actin (VEH: fold 1) are shown. Figures are representative of two independent experiments each one performed in duplicate. ^∗^*P* < 0.05; ^∗∗^*P* < 0.01; ^∗∗∗^*P* < 0.001 versus vehicle-treated cells (VEH).

Diverse cell survival signaling pathways are modulated by BCR-ABL1, such as JAK/STAT, MAPK, and PI3K/AKT/mTOR signaling pathways (Sinclair et al., 2013). Thus, we studied the effects of NPQ-C6 on BCR-ABL1-STAT5 signaling pathway. Constitutive phosphorylation of -STAT5 (pTyr^694^) and BCR-ABL1 (pTyr^177^) are crucial for K562 cell survival. Interestingly, both of them were significantly inhibited by NPQ-C6 (5 μM for 6 h) (Figure 4A). The inhibitory effect of NPQ-C6 on BCR-ABL1 was mainly due to reduced BCR-ABL1 protein content (Figure 4A). To further support the hypothesis that NPQ-C6 was able to inhibit constitutive pYSTAT5 in leukemia cells, we investigated the effects of NPQ-C6 on HEL, a human erythroleukemia cell line in which the JAK2/STAT5/STAT3 signaling pathway is constitutively activated due a JAK2 mutation (V617F) (Liu et al., 1999). Accordingly, NPQ-C6 also inhibited constitutive activation of pTyr^1007/1008^-JAK2 (pYJAK2), pTyr^705^-STAT3 (pYSTAT3) and pYSTAT5 (5 μM for 6 h) in HEL cells (Figure 4B). To analyze whether NPQ-C6 was also able to downregulate cytokine-induced STAT5/3 activities, we used a human breast adenocarcinoma cell line (T47D) which fails constitutively active STAT but is able to respond to GH and IL6, which are activators of STAT5 and STAT3 signaling pathway, respectively (Shiu and Paterson, 1984; Badache and Hynes, 2001). GH- or IL6-stimulated T47D cells were treated with NPQ-C6 at different times (5 μM for 0–3 h) and with different doses (0–5 μM for 30 min) and then examined for pYSTAT5 (Figure 5A) or pYSTAT3 (Figure 5B), respectively. As we previously reported (Guerra et al., 2017), GH induced pYSTAT5 within 10 min whereas IL6 induced pYSTAT3 within 30 min. Exposure of T47D cells to NPQ-C6 for 30 min (0.5 h) was sufficient to suppress GH-induced pYSTAT5 (Figure 5A) as well as IL6-stimulated pYSTAT3 (Figure 5B). Next, we decided to investigate the functional consequences of NPQ-C6-mediated inhibition of pYSTAT. To do that, firstly, we studied whether NPQ-C6 was capable of inhibiting STAT-dependent transcription. Two cell lines transfected with a luciferase reporter element were used to study STAT-dependent transcription, as described in Section “Materials and Methods.” NPQ-C6 was found to inhibit GH-STAT5 (IC50 = 7.9 ± 2.8 μM) and OMS-STAT3 (IC50 = 8.2 ± 0.1 μM) dependent transcription (Figure 6A) in HEK (Maamra et al., 1999) and HeLa cells (Stephens et al., 1998), respectively. Secondly, we evaluated the potential effect of NPQ-C6 on STAT-DNA interaction in GH or IL6-stimulated T47D cells. In correspondence with pYSTAT inhibition, NPQ-C6 suppressed GH- and IL6-induced binding to DNA of STAT5 (Figure 6B) and STAT3 (Figure 6C), respectively. Finally, functional consequences of NPQ-C6 on K562 cells were shown by decreased protein content of C-MYC and PIM-1 (Figure 6D), two target gene products of BCR-ABL1/STAT5 signaling pathway (Ren, 2005). These results indicate that NPQ-C6 is able to inhibit both constitutive as well as cytokine-induced activation of STAT signaling pathway. Next, we investigated the effects of NPQ-C6 on key signaling proteins in K562 cells. NPQ-C6 transiently increased the phosphorylation levels of JNK, P38-MAPK and AKT, whereas decreased the phosphorylation levels of ERK1/2 in comparison to the level of phosphorylation observed in vehicle-treated K562 cells (Figure 7). Activation of JNK, a protein kinase that is crucial for BCR-ABL1-induced cellular proliferation and transformation (Raitano et al., 1995), was observed as early as 1 h after NPQ-C6 treatment and continued for at least 6 h (Figure 7).

### NPQ-C6 Inhibits Cell Viability and Downregulates BCR-ABL1/STAT5 Signaling Pathway in IM-Resistant CML Cells

**FIGURE 8:**
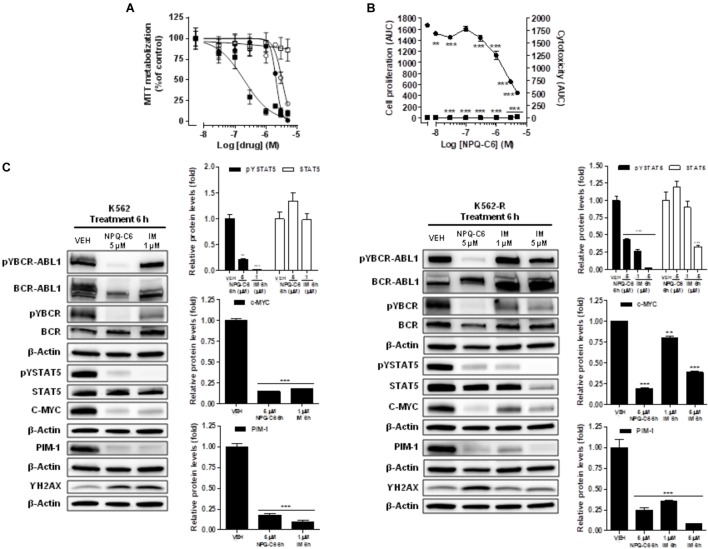
NPQ-C6 reduces viability and growth, and downregulates BCR-ABL1/STAT5 signaling pathway in imatinib-resistant K562 cells (K562-R). **(A)** IM-sensitive K562 cells (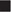, 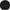) and IM-resistant K562-R (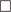, 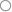) cells were cultured in the presence of increasing concentrations of NPQ-C6 (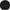, 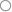) or IM (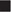, 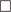) for 48 h. Cell viability was measured by the MTT assay. **(B)** K562 cells were cultured in the presence of vehicle or increasing concentrations of NPQ-C6 (0.03–5 μM) for 4 days. During that time the effects of the drug on proliferation (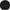, 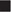) and cytotoxicity (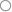, 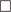) were investigated in K562 (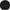, 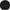) and K562-R (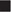, 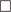) using the Incucyte^TM^ HD real-time analysis system. Data are represented as AUC. **(C)** K562 and K562-R cultures were incubated with vehicle (VEH), NPQ-C6 (5 μM) or IM (1 or 5 μM) for indicated times. Then, immunoblotting analyzes were performed to detect the phosphorylated and total levels of BCR-ABL1, BCR and STAT5, and to detect the protein levels of c-MYC, PIM-1, and γH2AX. Densitometric quantification of immunosignal values of phosphoproteins and total proteins relative to β-Actin (VEH: fold 1) are shown. Figures are representative of two independent experiments each one performed in duplicate. ^∗∗^*P* < 0.01; ^∗∗∗^*P* < 0.001 versus vehicle-treated cells (VEH).

To evaluate the width of the NPQ-C6 inhibitor, we investigated its activity against IM-resistant CML cells. The IM resistant K562-R (Aceves-Luquero et al., 2009) cells and the equivalent IM sensitive K562 cells were treated with increasing concentrations of NPQ-C6 or IM for 24–72 h and cell viability was analyzed by MTT metabolization assay. As we previously reported (Guerra et al., 2017), IC50 of IM needed for inhibit cell viability was 25 times higher in K562-R cells than in IM-sensitive K562 cells (Figure 8A). However, both K562 as well as K562-R cells showed equivalent sensitivity to NPQ-C6, with IC50 values close to 2–3 μM for both clones (Figure 8A). Live-Cell imaging of K562 cells confirmed that NPQ-C6 caused cytostatic or cytotoxic effects on IM sensitive as well as K562-R cells with IC_50_ values close to 2–3 μM, respectively (Figure 8B). To test the hypothesis that NPQ-C6 was able to maintain its molecular mechanism of action in both cell clones, we next investigated the effects of this product on relevant signaling pathways implicated in the product-mediated effects on K562-R cells (Figure 8C). NPQ-C6 (5 μM for 6 h) caused similar inhibition of pYBCR-ABL1, pYBCR, pYSTAT5, C-MYC, PIM-1 and stimulation of γH2AX in both cell clones (Figure 8C). However, IM required concentrations up to 5 μM (6 h) in K562-R cells to induce similar effects on the commented signaling proteins compared to its IM-sensitive counterpart (Figure 8C).

### Molecular Modeling Predicts That BCR-ABL1 and JAK2 Proteins Are NPQ-C6 Targets

**FIGURE 9:**
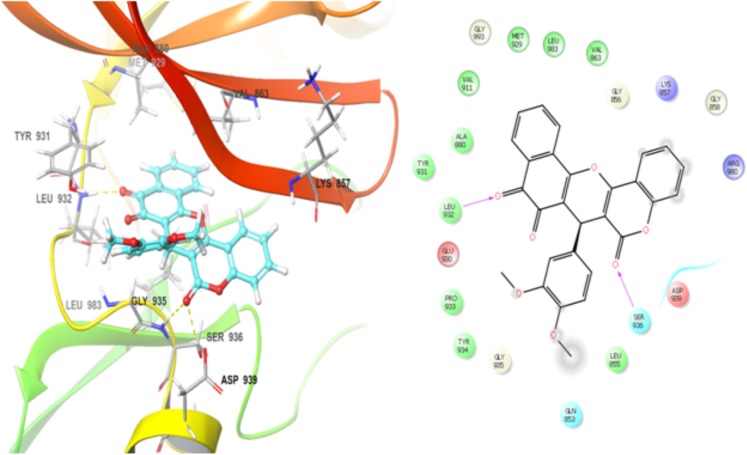
The binding mode of NPQ-C6 to the kinase catalytic domain of the JAK2 protein. NPQ-C6 was docked into the kinase catalytic domain of JAK2 and its binding pattern was analyzed by flexible molecular docking as described into Section “Materials and Methods.” The highest docking scores values were ranged from 8.32 to 10.1 kcal mol^-1^. The existence of two hydrogen bonds can be observed: first, between Leu 932 and the carbonyl group of the NPQ nucleus and second, between Ser 936 and the carbonyl group of the coumarin moiety. Hydrophobic interactions between the aromatic ring of the coumarin-NPQ moieties and others amino acids (i.e., Gln 853, Val 863, Leu 855, Lys 857, Leu 983, Ala 880, Asp 939, Gly 993, Met 929, Tyr 931, Gly 935) are also observed.

BCR-ABL1 and JAK2 proteins regulate STAT5 phosphorylation and these proteins are drug targets for therapeutic intervention in leukemia (Ren, 2005; Warsch et al., 2013). Thus, NPQ-C6 was docked into the kinase catalytic domain of BCR–ABL1 and JAK2 in order to propose the possible mode of action and establish the structural determinants and key interactions responsible for the inhibitory activity (Eldridge et al., 1997; Friesner et al., 2004). Docking results showed that NPQ-C6 bound to the Abl binding site in the hinge region by making hydrophobic interactions between the aromatic rings of NPQ-C6 and hydrophobic residues present in the ATP domain. Particularly, two edge-to-face π-π stacking interactions were found between the quinone aromatic ring and Tyr 253, and between the aromatic ring of the coumarin moiety and Phe 317. Other hydrophobic interactions with Met 318, Met 290, Leu 248, Leu 370, and Leu 248 were also observed. This type of interactions could be responsible of the observed high inhibitory activity toward BCR-ABL1 kinase. Notably, the best docking score value for the best conformation was -6.12 kcal mol^-1^. An exhaustive analysis of the docking results shows that NPQ6 fitted better into the JAK2 ATP-binding site. The binding pattern of NPQ-C6 was analyzed by flexible molecular docking (LigPrep, 2017) and the highest docking scores values were ranged from -8.32 to -10.1 kcal mol^-1^. NPQ-C6 showed a good steric and electronic complementarity with the ATP-binding site, being observed the existence of two hydrogen bonds (Figure 9): 1) between the residue Leu 932 and the carbonyl group of the NPQ nucleus, and 2) between Ser 936 and the carbonyl group of the coumarin moiety. Hydrophobic interactions between the aromatic ring of the coumarin-NPQ moieties and other amino acids (i.e., Gln 853, Val 863, Leu 855, Lys 857, Leu 983, Ala 880, Asp 939, Gly 993, Met 929, Tyr 931, and Gly 935) were also observed (Figure 9).

## Discussion

The NPQ- and coumarin-based derivatives represent promising scaffolds in medicinal chemistry for finding novel inhibitors of carcinogenic pathways (Hsu et al., 2006; Medina et al., 2015; Zhao et al., 2015; Guerra et al., 2017; Hueso-Falcon et al., 2017). These are exemplified by quinone-based antitumor agents (e.g., doxorubicin) which are clinically used to treat solid tumors or acute lymphoblastic leukemia. Interestingly, it has been shown that coumarin-chalcone hybrids reduce cell proliferation and induce apoptosis in K562 cells (Elshemy and Zaki, 2017). However, the potential effects of NPQ-coumarin based compound on CML have not been previously investigated. In this study, NPQ-C6, a novel NPQ-coumarin hybrid compound, was shown to inhibit BCR-ABL1/STAT5/c-MYC/PIM-1 oncogenic pathway in both IM-sensitive and IM-resistant CML.

NPQ-based derivatives exhibit pharmacological activities by a number of mechanisms, including oxidative stress, Michael-type arylation of thiol groups in biomolecules, DNA intercalation and bioreductive alkylation *via* quinone methide formation, autophagy, apoptosis, inhibition of topoisomerases, cell cycle arrest, or inhibition of c-MYC and BCR-ABL1/STAT5 pathway (Hsu et al., 2006; Zhao et al., 2015; Guerra et al., 2017; Hueso-Falcon et al., 2017). In this study, we demonstrated that NPQ-C6 induces apoptosis in K562 cells as indicated by increased annexin V binding, cleavage of caspase-3, -9, and PARP and induced caspase-3, -9 activities. These findings suggest that NPQ-C6 triggered the mitochondrial apoptotic pathway (Danial and Korsmeyer, 2004). Paradoxically, Z-VAD, a pan-caspases inhibitor, did not prevent NPQ-C6- reduced viability of K562 cells. Notably, NPQ-C6 augmented double-strand DNA break marker γH2AX which points out that K562 cells cannot overcome cell cycle arrest and they are destined for apoptosis. Similarly, it has been reported that some chemotherapy agents increased expression of γH2AX, DNA damage and apoptosis (Danial and Korsmeyer, 2004; Wu et al., 2015; Guerra et al., 2017). The effects of NPQ-C6 on K562 cells involved cell cycle arrest with increased of sub-G1 and reduced G0/G1 cell cycle phases. Similarly, CM363, a NPQ-based product, has been shown to induce cell cycle arrest through increased S phase and reduced G0/G1 and G2/M phases (Guerra et al., 2017). These effects were related to changes on expression levels and/or phosphorylation of many proteins involved in cell cycle progression (Guerra et al., 2017). Interestingly, several of the most potent CDC25 inhibitors are, similarly to NPQ-based derivatives, quinone-containing compounds (Brenner et al., 2014). However, if a quinone- or quinone-coumarin associated mechanism or changes on activities of proteins involved in cell cycle progression are linked to DNA damage and apoptosis deserves future research.

Inhibition of BCR-ABL1/STAT5 signaling pathway in CML cells can induce apoptosis (Berger et al., 2014). Accordingly, cell cycle arrest and apoptosis by NPQ-C6 were related to BCR-ABL1/STAT5 downregulation, an effect previously shown for NPQ-based derivatives (Guerra et al., 2017). Functional consequences of BCR-ABL1/STAT5 pathway inhibition by NPQ-C6 were shown by decreased protein content of c-MYC and PIM-1, two gene products connected with BCR-ABL1/STAT5 signaling pathway (Ren, 2005). Interestingly, MYC, a transcription factor belonging to the basic-helix-loop-helix-leucine zipper (bHLH-LZ) family, has been implicated in BCR/ABL1 mediated transformation (Hoffman et al., 2002). Furthermore, it has been shown that BCR/ABL1 can induce c-MYC activity through distinct mechanisms, including regulation of c-MYC expression mediated by PI3K and JAK2 pathways (Xie et al., 2002), E2F1-induced inhibition of c-MYC proteasome-dependent degradation through activated JAK2 (Stewart et al., 1995), and modulation of MYC mRNA translation (Notari et al., 2006). Relevant, c-MYC can lead to up-regulation of BCR/ABL1 protein level (Sharma et al., 2015), which is critical for CML progression from chronic phase to blast crisis (Perrotti et al., 2010). Notably, BCR/ABL1 tyrosine kinase activity and expression can be reduced by pharmacological inhibition of MYC (Lucas et al., 2015). MYC inhibition leads to reduced proliferation and induction apoptosis (Sharma et al., 2015). Importantly, our study demonstrates that NPQ-C6 decreased PIM-1 protein content, a member of PIM family of serine/threonine kinases which promote cell cycling, proliferation, and cell survival in hematological malignancies (Saurabh et al., 2014). This is exemplified by PIM-mediated regulation of c-MYC-dependent transcription (i.e., MCL-1) and oncogenic transformation (Zippo et al., 2007). Relevant to this study, targeting PIM kinases may provide a unique therapeutic approach for the treatment of BCR-ABL1^+^ leukemia (Curi et al., 2015). Although, direct effects of NPQ-C6 on c-MYC/PIM-1 regulated signaling have not been explored, our study suggests that inhibition of BCR-ABL1/STAT5/c-MYC/PIM-1 pathway by NPQ-C6 contributes to K562 cell death. The progress of CML involves not only the BCR-ABL1/STAT5/MYC/PIM signaling but also other survival pathways such as JNK, ERK 1/2, p38MAPK or AKT (Ren, 2005; McCubrey et al., 2008). The balance between the ERK1/2 cascade and p38MAPK and JNK pathways is a key event in the regulation of cell survival. ERK1/2 in most cases prevents apoptosis (Xiao et al., 2013), whereas the JNK and p38 have generally been related to pro-apoptotic events (Cross et al., 2000). However, p38MAPK signaling has also been shown to promote survival, cell growth and differentiation. In consequence, cell type and stimuli can influence the role of p38MAPK in apoptosis (Zarubin and Han, 2005). In the current study, we observed that NPQ-C6 was able to inhibit BCR-ABL1, resulting in a downregulation of ERK1/2 and p38MAPK phosphorylation whereas it caused a transient phosphorylation of JNK and AKT. These findings suggest that the effects of NPQ-C6 on the mentioned signaling pathways may be contributing in the mechanism by which the product activates apoptosis in K562 cells. AKT activation is a relevant event for survival and leukemogenesis (McCubrey et al., 2008; Kharas et al., 2010). Therefore, it might be speculated that the paradoxical transient activation of AKT phosphoryation induced by NPQ-C6 treatment may reflect an attempt of K562 cells to overcome BCR-ABL1 inhibition in order to promote survival. However, this hypothesis seems unlikely because NPQ-C6-mediated activation of AKT phosphorylation precedes inhibition of BCR-ABL1/STAT5 induced by the product. Another plausible explanation for this phenomenon might be that the product inhibits a negative AKT regulator, such as phosphatase and tensin homolog (PTEN), as it has been reported for the coumarin derivative fraxetin in immortalized human keratinocytes HaCaT cell line (Kundu et al., 2016). Further experiments are needed (e.g., combinatorial assays of PI3K inhibitors and NPQ-C6) in order to investigate the potential role of this rapid paradoxical transient AKT activation on the anti-tumor effects of this product in CML. Recent studies suggest that NPQ-based derivatives (e.g., shikonin, CM363) induced activation of JNK followed by apoptosis mediated by intrinsic signaling pathway in K562 cells (Hsu et al., 2006; Zhao et al., 2015; Guerra et al., 2017). In the present work, we have shown that NPQ-C6 inhibits BCR-ABL1/STAT5 and promotes JNK phosphorylation, so we cannot rule out the possibility that both mechanisms contribute to NPQ-C6-induced apoptosis. Reduced cell viability caused by NPQ-C6 treatment was also associated with decreased levels of BCR-ABL1 protein which suggests that the mechanism of NPQ-C6 action in K562 cells was connected, directly or indirectly, to protein synthesis inhibition of protein, as it has been described for other NPQ-based derivatives (Guerra et al., 2017).

Resistance to TKI drugs is a major challenge to CML chemotherapy and efforts on finding novel drugs for use as co-adjuvant for conventional TKI therapy must be done (Druker et al., 2006; Quintas-Cardama et al., 2009). Related to the later, several mechanisms of resistance have been reported, including amplification or mutation of the BCR-ABL1 gene, drug efflux or activation of JAK2-STAT5 signaling pathway (Druker et al., 2006; McCubrey et al., 2008; Quintas-Cardama et al., 2009; Quintas-Cardama and Verstovsek, 2013). Importantly, despite the mechanism through which NPQ-C6 circumvented IM-resistance was not explored, NPQ-C6 inhibited pYBCR-ABL1, pYSTAT5, c-MYC or PIM-1 in K562-R cells, which suggests that NPQ-coumarin conjugates might be able to circumvent resistance to TKI in BCR-ABL1^+^ leukemia.

Finally, docking analysis showed that NPQ-C6 binds to the Abl binding site in the hinge region by making hydrophobic interactions between the aromatic rings of the hybrid compound and some hydrophobic residues present in the ATP domain which could be responsible of the inhibitory activity toward BCR-ABL1 kinase. Particularly, two edge-to-face π-π stacking interactions were found between the quinone aromatic ring and Tyr 253, and between the aromatic ring of the coumarin moiety and Phe 317. In addition, NPQ-C6 fit better into the JAK2 ATP-binding site. This compound showed a good steric and electronic complementarity with the binding site, being observed the existence of two hydrogen bonds, one of them between the residue Leu 932 and the carbonyl group of the NPQ nucleus, as well as another hydrogen bond between Ser 936 and the carbonyl group of the coumarin moiety which are similar to those observed for known JAK2 inhibitors (Zak et al., 2013; Hart et al., 2015).

In summary, the present study shows for the first time that the NPQ-coumarin conjugate NPQ-C6 is a multi-targeting agent which has potent anti-CML effects. Reduced survival of K562 cells after exposure to NPQ-C6 is associated with induction of JNK activity, cell cycle arrest, apoptosis and inhibition of BCR-ABL1/STAT5/c-MYC/PIM-1 signaling pathway. Clinically relevant, NPQ-C6 mantains its antitumoral activity and molecular mechanism of action in IM-resistant CML cells. Although more research is needed to investigate *in vivo* antitumor efficacy and toxicity, these data suggest that the development of novel NPQ-C6-based derivatives may allow the identification of therapeutic agents active against BCR-ABL1^+^ leukemia, even in those cases in which IM resistance is already established.

## Author Contributions

BG, JD-C, JQ, FE, AA, AE-B, and LF-P conceived and designed the experiments. PM-R, BG, IH-F, AA, and HA-T performed the experiments. BG, JD-C, JQ, FE, AE-B, BD-C, and LF-P analyzed the data. BG, AE-B, and LF-P wrote the paper.

## Conflict of Interest Statement

The authors declare that the research was conducted in the absence of any commercial or financial relationships that could be construed as a potential conflict of interest.

## Footnotes

1 www.graphpad.com
